# Endotheliitis, Shunts, and Ventilation–Perfusion Mismatch in Coronavirus Disease 2019: A Literature Review of Disease Mechanisms

**DOI:** 10.1016/j.amsu.2022.103820

**Published:** 2022-05-17

**Authors:** Francisco J. González-Ruiz, Emmanuel A. Lazcano-Díaz, Luis A. Baeza Herrera, Montserrat Villalobos-Pedroza, Enma L. Toledo Alemán, Miriam G. Zuñiga-Salcedo, Camelia Cruz-Rodríguez, Alexandra López-Polanco, Abraham Torres-Pulido, Alejandro Sierra-González de Cossio, Luis A. Cota Apodaca, Daniel Manzur-Sandoval

**Affiliations:** a Department of Cardiovascular Critical Care, National Institute of Cardiology “Dr. Ignacio Chávez,”, Mexico City, México; b Department of Cardiovascular Diseases, National Institute of Cardiology “Dr. Ignacio Chávez,”, Mexico City, Mexico

**Keywords:** Endotheliitis, Shunt, V/Q mismatch, HPV mechanism, COVID-19

## Abstract

The severe acute respiratory syndrome coronavirus 2 pandemic has continued to impact global health. However, while immunity acquired by vaccines has been developed, 40% of the world's population has still not been vaccinated. Economic problems associated with acquiring novel therapies, misinformation, and differences in treatment protocols have generated catastrophic results, especially in low-resource countries. Understanding the pathophysiological aspects of coronavirus disease and the therapeutic strategies that have been validated to date is essential for successful medical care. In this review, I summarize the historical aspects of the virus, molecules involved in infecting the host, and consequences of viral interactions with and in tissues.

## Highlights

• 1.- The viral interaction with multiple structural proteins explains to a great extent the diversity of the different routes of viral access to the organism.
• 2.- The involvement of the various receptors seems to broadly explain the constellation of clinical manifestations seen in affected patients.
• 3.- The “silent hypoxemia,” which has captured much of the attention in the scientific community, is primarily explained by mechanisms that include intrapulmonary anastomoses, shunt, and perfusion alterations as a whole and not individually.
• 4.- Efforts aimed to understand the pathophysiological bases of SARS-CoV2 disease could improve the understanding of clinical manifestations of the disease, contribute to effective treatment, and develop new technologies that allow viral non-interaction with the organism.

## Abbreviations

**ACE2**: angiotensin-converting enzyme 2
**ARDS**: acute respiratory distress syndrome
**COVID-19**: coronavirus disease
**FiO**_**2**_: fraction of inspired oxygen
**HPV**: hypoxic pulmonary vasoconstriction
**IBA**: intrapulmonary bronchopulmonary anastomoses
**IFNs**: interferons
**MERS**: Middle East respiratory syndrome
**NO**: nitric oxide
**NRP1**: neuropilin 1
**PaO**_**2**_: partial pressure of oxygen
**PEEP**: positive end-expiratory pressure
**RNA**: ribonucleic acid
**SARS-CoV-2**: severe acute respiratory syndrome coronavirus 2
**TMPRSS2**: transmembrane serine protease 2
**VSELs**: very small embryonic stem cell

## 1 Introduction

The 2019 coronavirus disease pandemic, which began in China in 2019, continues, with a cumulative death toll exceeding five million worldwide. The heterogeneous descriptions described in clinical symptoms and paraclinical and different phenotypes of the disease suggest a variety of pathophysiological aspects involved in this entity, which differ significantly from those presented in other forms of respiratory distress syndrome.

Understanding the origin of coronaviruses, their characteristics, and the scientifically proven evidence during this pandemic shed light on the pathophysiological aspects of the disease, which should not be left aside for the establishment of therapeutic strategies.

The approach carried out in this review covers the historical perspective of the disease and the analysis and interpretation of the pathological aspects of the disease, which distinguish covid-19 infection from other similar diseases.

The published information is extensive, so we try to take the outstanding evidence available today and summarize it simply for the reader, which allows for a better understanding of the disease process and timely medical care of infected patients.

## 2 Historical perspective of the coronavirus

### 2.1 Process of viral evolution to the present day

Coronaviruses are microorganisms that have been identified for almost a century [1]. They belong to the Coronaviridae family of the order Nidovirales [2]. The viral surface has crown-shaped peaks, which are minuscule (65–125 nm in diameter). The first reports of diseases associated with these agents were described in 1930 when cases of respiratory infections derived from poultry were identified, which were later classified as gammacoronavirus [3,4]. In the 1950s, cases of porcine transmissible gastroenteritis appeared, identifying the infectious agent as an alphacoronavirus [5,6]. However, it was not until 1960 that the first cases of coronavirus infection were identified in humans during the isolation of tracheal organ cultures [6]. The concern of the scientific community soared when, in 2003, an outbreak of severe acute respiratory syndrome coronavirus (SARS-CoV) secondary to a zoonotic agent caused severe acute respiratory syndrome (SARS) [7,8], which was quickly controlled, and strains of HcoV-NL63 and HcoV-HKU1 were identified, which are considered community respiratory coronaviruses [9,10]. Close monitoring of these agents continued, with a second outbreak in 2012, causing Middle East respiratory syndrome (MERS) [7,8], with beta coronavirus being the agent responsible for this health emergency. To date, MERS has continued to appear in our environment, with a low profile and limited spread.

## 3 Route of entry and Endotheliiitis

### 3.1 Viral morphology, entry proteins, and initial immune response

**Fig. 1:**
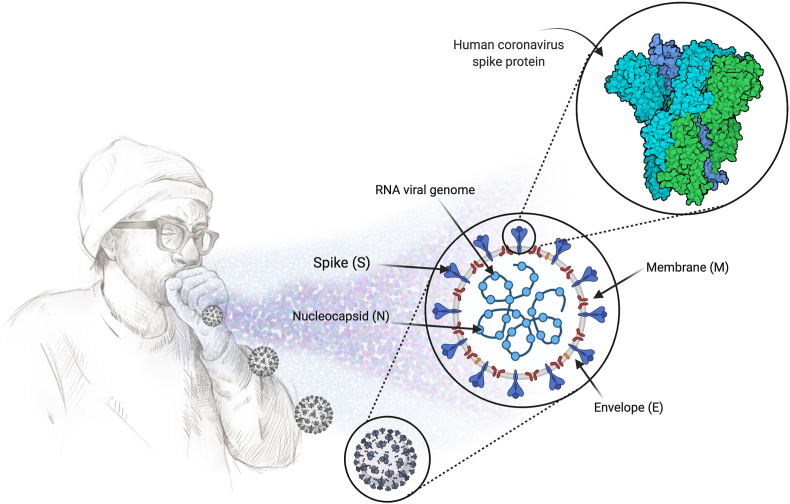
Potential transmission mechanism and distinctive morphological characteristics of SARS-CoV-2.

**Fig. 2:**
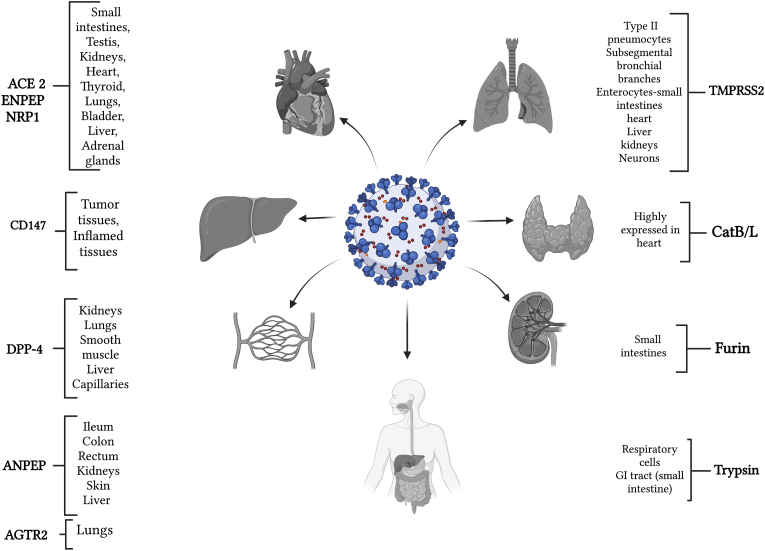
- Known mechanisms of viral entry: Receptors (left) and Proteases (right). ACE2: Angiotensin-converting enzyme 2; ENPEP: glutamyl aminopeptidase; NRP1: Neuropilin-1; DPP4: Dipeptidyl peptidase; ANPEP: alanyl aminopeptidase; AGTR2: angiotensin II receptor type; TMPRSS2: Transmembrane serine protease 2; Cat B/L: Cathepsin B and L. (Adapted from *Masre SF, Jufri NF, Ibrahim FW, Abdul Raub SH. Classical and alternative receptor for SARS-CoV-2 therapeutic strategy. Rev Med Virol. 2021;31(5):e2207.*https://doi.org/10.1002/rmv*.2207)*.

Coronavirus disease 2019 (COVID-19) spreads via respiratory droplets and aerosols during person-to-person contact [11]. This agent has distinctive morphological characteristics: four structural proteins: peak protein (S), membrane (M), envelope (E), and nucleocapsid (N) [11]. Protein S, responsible for host penetration, is probably the most complex of all viral glycoproteins and plays a crucial role in immune responses [[11], [12], [13]]. The SARS-CoV-2 peak glycoprotein represents SARS-CoV in bats and an unknown beta-CoV [14]. Protein S contains a receptor-binding domain that explicitly recognizes angiotensin-converting enzyme 2 (ACE2) [14] (Fig. 1). Simons and Helenius first described entry mechanisms [15] using electron microscopy in the 1980s. In addition to glycoprotein S, non-specific binding factors (ACE2, dipeptidyl peptidase 4, alanyl aminopeptidase, and glutamyl aminopeptidase) are also included in its structure [15]. Later, Matsuyama et al. [16] demonstrated that membrane proteases are elements produced by lung inflammatory cells (elastase) and activating cells of protein S (transmembrane serine protease 2 [TMPRSS2]) that lead to greater efficiency in the infectious process, thereby causing severe damage in patients. Shulla et al. [17] subsequently showed that the presence of the viral receptor (ACE2) and activating proteases (TMPRSS2) is required in the cell plasma membrane and is a fundamental step in the spread of infection. Recent studies [18] have reported the involvement of proteases in viral activation (ACE2 and TMPRSS2) in hematopoietic precursors and endothelial cells, known as very small embryonic stem cells (VSELs). The ACE-SARS-CoV2 interaction in VSELs leads to inflammasome activation. These inflammasomes represent a protein complex responsible for the cascade of inflammatory events that leads to cell death due to pyroptosis [18]. (Fig. 2).

A mutation in N501T has also been identified in the SARS-CoV-2 spike protein, which could significantly improve the affinity for ACE2 binding; this has not been previously identified in other types (SARS-CoV) [19].

Once membrane fusion is performed, viral entry into alveolar epithelial cells is initiated. The viral content is released, the replication process begins, and ribonucleic acid (RNA) is subsequently formed by RNA polymerases [20,21]. Negative-strand RNA is a vital element that produces new strands of positive RNA that later synthesizes new proteins in the cell cytoplasm [20,21]. The role of the remaining glycoproteins is not less important. Viral protein N binds to genomic RNA. Protein M generates a favorable environment for integration into the endoplasmic reticulum [22]. These complexes (nucleocapsids) are lodged in the endoplasmic reticulum membrane. They are transported to the lumen, sent to Golgi vesicles in the cell membrane, and exocytosis occurs in the extracellular space. Thus, the viral particles are ready to invade adjacent epithelial cells, which provides the infectious material for community transmission [22].

The ACE2 receptor in cells is the main factor for host entry and is highly expressed in the epithelial cells of the adult respiratory tract [23,24]. The virus then undergoes local replication, spread, and infection of hairy cells. This process lasts for a couple of days, and despite the low viral load, the carrier is highly infectious [24]. The carrier experiences mild symptoms, characterized by fever and malaise, during this phase. At this stage, the immune response is mediated by chemokine ligand 10 of the CXC motif (CXCL-10) and interferons (IFNs) from infected cells (IFN-β and IFN-λ) [25]. It is essential to highlight that, although ACE2 has been considered the entry receptor in adults, alternative receptors for this virus have been proposed to explain the infectious nature in a variety of particularly susceptible groups. Some alternative receptors that facilitate host entry include neuropilin and CD209L, which are highly expressed in endothelial cells [26].

The CD209L receptor, which represents the pathway of SARS-CoV infection, is a type C lecithin transmembrane glycoprotein expressed in type II pneumocytes and pulmonary endothelial cells [26]. Immunofluorescence studies in human tissues showed high expression of CD209L in the endothelium of small and medium vessels, which prevail in the lungs and kidneys [26,27]. Neuropilin 1 (NRP1) recognizes and binds to the peak protein of SARS-CoV-2, and its upward expression has been identified in blood vessels infected with SARS-CoV-2 [28]. NRP1 is associated with biological processes, including the development of the cardiovascular system, angiogenesis, and neuronal circuits, and is involved in the penetration of the virus into the central nervous system [28,29].

Once in alveolar epithelial cells, the virus initiates replication and increases the number of viral nucleocapsids [28,29]. Alveolar cells initiate the release of multiple cytokines and inflammatory mediators (interleukin [IL]-1, IL-6, IL-8, IL-20, and IL-12), tumor necrosis factor, monocyte chemoattractant protein-1, and macrophages-1α [30,31]. This release of cytokines initiates the “cytokine storm” and acts as a chemoattractant for inflammatory cells sequestered in lung tissues [32,33]. The immune response is responsible for inflammation and lung injury when the virus fights. Apoptosis of infected cells releases new viral particles [34,35]. Repetitive injury, together with viral replication, leads to the loss of type 1 and 2 pneumocytes, which generates diffuse alveolar damage that ultimately culminates in acute respiratory distress syndrome (ARDS) [34,35].

The vascular endothelium plays a fundamental role, represents a very efficient barrier between the circulation and adjacent tissues, and has critical functions in maintaining vascular tone, hemostasis, inflammatory response, coagulation, and a component of the extracellular matrix [36]. Therefore, excessive systemic inflammation suggests a critical pathophysiological role in the presentation and progression of COVID-19 [[37], [38], [39], [40]].

**Fig. 3:**
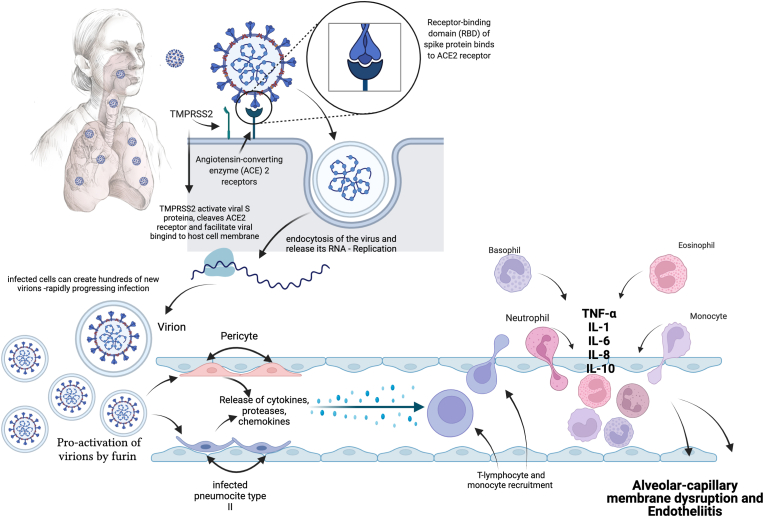
- Proposed mechanism of endotheliitis. The endothelium maintains homeostasis, regulating systemic blood flow tissue perfusion in conjunction with smooth muscle cells and pericytes, promoting vasodilation, regulation of vascular permeability, and controlling antithrombotic and immune effects. Viral infection primarily affects vascular endothelial cells, epithelial cells, and pulmonary macrophages, causing hyper inflammation and attenuated antiviral responses. There is evidence for the pre-activation of virions by the proprotein convertase Furin during entry into host cells. Dysregulation in the renin-angiotensin-aldosterone system and the bradykinin-kallikrein pathway leads to downregulation of ACE2, resulting from ADAM17-mediated seeding. Angiotensin II promotes microvascular thrombosis, coagulopathy, and hypofibrinolysis, subsequently recruiting inflammatory mediators.

Endotheliitis represents a vascular inflammation that separates the basement membrane from the subendothelial lymphocytes, and is the most important hallmark of viral infection. Additionally, histopathological findings showed diffuse endothelial inflammation and apoptosis [41]. Further evidence for the fundamental role of the endothelium in the pathophysiology of the disease includes significantly elevated levels of factor VIII and von Willebrand factor in some patients infected by the virus; these patients develop cerebrovascular events without any other manifestation [[41], [42], [43]]. (Fig. 3).

**Fig. 4:**
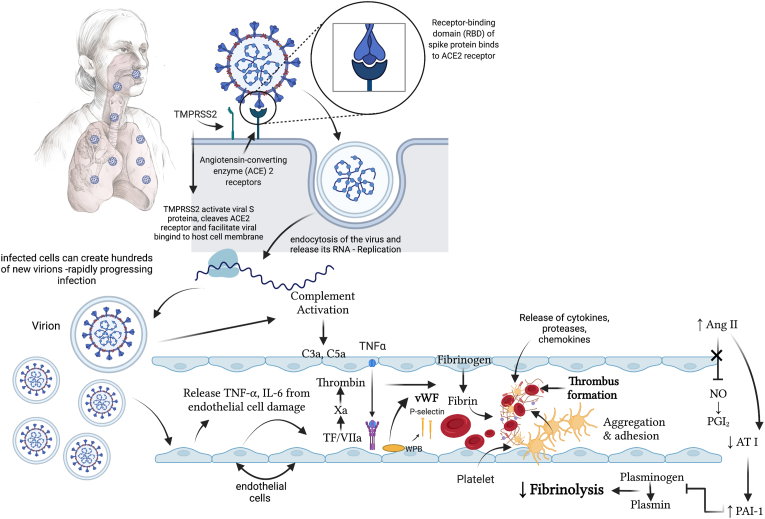
Immunothrombosis mechanisms. As previously mentioned, endothelial dysregulation, with the subsequent increase in Ang II, promotes microvascular thrombosis, coagulopathy, and hypofibrinolysis. The increase in the release of inflammatory cytokines, TNF-α and IL-6, due to direct or indirect endothelial damage mediated by SARS-CoV-2 and viral persistence, exacerbates a hyperinflammatory state. Additionally, the coagulation cascade is activated, secondary to the release of tissue factor with induction of a prothrombotic state. There is evidence that SARS-CoV-2 promotes complement activation, increasing the release of anaphylatoxins (C3a, C5a), which improve immune cell recruitment, perpetuate endothelial damage, and announce the release of reactive oxygen species. Fibrinolysis is inhibited by Ang II, mediated by the increase in PAI-1. TNF-α: tumor necrosis factor-alpha; IL-6: interleukin-6; TF: tissue factor: Ang II: angiotensin II; NO: Nitric Oxide; PGI_2_: Prostaglandin I2; AT-I: angiotensin receptor subtype I; PAI-1: Plasminogen activator inhibitor-1, WPB: Weibel-Palade Bodies; vWF: Von Willebrand factor.

Therefore, endothelial dysfunction and subsequent endotheliitis result from the direct endothelial invasion of SARS-CoV, leading to endothelial inflammation and leukocyte recruitment. These trigger inattentive immune responses and are responsible for microthrombotic complications. More evidence of endothelial involvement was shown in a study that found extensive endothelial and subendothelial complement deposits (C4d and C5b-9) in the affected vessels and peak and viral envelope glycoproteins [[40], [41], [42], [43], [44]] (Fig. 4).

Other effects of endothelial dysfunction associated with viral infection include biosynthesis of nitric oxide (NO), which is responsible for vasodilation, angiogenesis, and proliferation of endothelial cells. NO availability depends on the balance between reactive oxygen species production and endothelial nitric oxide synthase activity [45]. Increased oxidative stress and decreased NO availability and synthesis lead to superoxide generation [45,46].

The diversity in the mechanisms of viral entry could explain the significant differences in the manifestations between symptomatic and asymptomatic carriers and the severity between different groups of vulnerable patients.

## 4 Shunts and COVID-19

### 4.1 Mechanisms of hypoxemia: intrapulmonary anastomoses and intussusceptive angiogenesis

Silent hypoxemia is undoubtedly one of the distinctive features of COVID-19. However, the underlying mechanisms are not fully understood, especially in patients with preserved lung elastance [47]. Preserved lung elastance in mechanically ventilated patients with severe pneumonia reflects an increased lung dead space and a predominant perfusion defect. Dead space in these patients may be due to vasoconstriction or prevalent micro- or macrothrombosis; therefore, the use of vasodilators and anticoagulants would play a leading role [47].

Several studies have reported the presence of patent foramen ovale and transpulmonary bubbles during contrast echocardiography, with a prevalence of 10–20% among infected patients. Although it was first considered the basis for the explanation of silent hypoxemia, it cannot be entirely attributed to the existence of short circuits [48]. The presence of transpulmonary bubble transit is not exclusive to COVID-19, and its prevalence in other forms of ARDS is estimated to be up to 26% [48,49]. The presence of thrombosis, apoptosis, edema, inflammation, and angiogenesis contributes to the dysregulation of the pulmonary vasculature, which leads to perfusion alterations and explains the heterogeneity of the reported phenotypes.

Additional investigations have reported the presence of prominent intrapulmonary bronchopulmonary anastomoses (IBAs) between the pulmonary and bronchial arteries as a potential source of a right-left shunt, which is not exclusive to COVID-19 infection and has been reported in idiopathic pulmonary hypertension and chronic thromboembolic pulmonary hypertension [50]. These IBAs predominate in fetal life and are obliterated at birth, but may persist in the context of the disease [51,52]. These are vascular connections with diameters varying between 15 and 500 μm. They are characterized by their ability to divert deoxygenated blood that bypasses the pulmonary microvascular bed, leading to alterations in distal pulmonary perfusion and representing gas exchange sites [51,52]. These prominent anastomoses explain the microanatomic substrate associated with hypoxemia in patients with COVID-19 in a better way [53]. This is clinically relevant because shunts reduce arterial oxygen saturation through arteriovenous mixing and do not respond to increased fractions of inspired oxygen (FiO_2_) [53]. Trials performed have correlated the presence of these shunts with increases in C-reactive protein and dehydroleucodine levels, and the severity and number of shunts associated with an adverse prognosis. Similarly, the mechanism of shunts better explains the dependence of prone decubitus on oxygenation by improving blood redistribution [53].

The effects of previously addressed endotheliitis and the presence of large shunts with secondary vascular dysregulation are also convincing explanations for the significant hypoxemia that appears early in the hypoxemic phase of the disease.

Dysregulation of the pulmonary vasculature is related to perfusion abnormalities and includes dilated peripheral vessels with a “tree-in-bud” configuration. The prevalence of these dilated vessels significantly increases the duration of hospitalization and ventilation [53]. The formation of these new vessels can occur in a conventional manner or by a mechanism of intussusceptive angiogenesis [54]. The underlying inflammatory process has been shown to induce recruitment of bronchopulmonary anastomoses, with the right-left shunt preventing the alveolar-capillary network and impairing gas exchange [55,56]. This shunt further reduces distal perfusion, leading to intractable hypoxemia and death [50]. Viral endocytosis reduces the availability of ACE2 and angiotensin II as regulators, which in this condition has a limited vasoconstrictor potential and downregulates ACE (cleavage effect mediated by the protease ADAM 17) [57]. The latter converts angiotensin I to angiotensin II and is responsible for the degradation of bradykinin, which has vasodilatory functions and may be involved in the worsening of shunts [57,58].

This vascular dysregulation abolishes the hypoxic pulmonary vasoconstriction mechanism, causing an increase in venous mixing, excessive pulmonary vasoconstriction, and micro-and macrothrombosis, with a consequent increase in dead space [57,58].

It is challenging to identify shunts to visualize their typical presentation as pulmonary involvement progresses [[59], [60], [61]]. Current consolidations obscure shunts, limit CO_2_-induced ventilation mechanism, and aggravate hypoxemia. Finally, this interaction between the pulmonary shunt that generates hypoxia and the hypercapnic ventilatory inhibition clarifies hypoxemia without increased respiratory effort or even without dyspnea, referred to in the literature as “silent hypoxemia,” in a better way [[61], [62], [63]].

## 5 Ventilation to perfusion (V/Q) mismatch in COVID-19

### 5.1 High and low V/Q ratio and hypoxic pulmonary vasoconstriction

The distinctive features of classic ARDS are similar to those of SARS-CoV-2, including diffuse alveolar damage, local edema with protein leakage, hyaline membranes, and hyperplasia of type II alveolar cells. However, severe endothelial injury and an increase in the prevalence of thrombotic phenomena may explain the accentuated effect on the pulmonary circulation. These alterations in local perfusion can also explain hypoxemia [64]. An increased ratio of perfusion to ventilation (V/Q > 1) and shunt (V/Q = 0) is not exclusive to the deterioration of hypoxic pulmonary vasoconstriction; however, redistribution of flow to vascular obstruction and restoration of tone is a challenge [[64], [65], [66], [67]].

It has been previously reported that in ARDS induced by COVID-19, the presence of short circuits that force redistribution and overflow of normally aerated units, in addition to interstitial edema that increases pulmonary vascular pressure with embolized units, leads to hypoxemia with differences in V/Q [67,68]. Capillary microthrombosis demonstrated in an autopsy study showed extensive intraseptal capillary thrombosis, leading to an increase in dead space and V/Q mismatch [67,68].

A low V/Q ratio (areas of high perfusion with poor ventilation) and intrapulmonary shunting are caused by alterations in respiratory mechanics, vascular obliteration, and alveolar infiltration, as well as impaired perfusion, which worsens if the coexistence of more of these persists in the same clinical entity, distinctive of COVID-19 [68].

Intrapulmonary shunts caused by pulmonary capillary distention or the presence of intrapulmonary arteriovenous anastomoses are closely related to the hyperdynamic state of patients with ARDS [69]. This has clinical relevance, as up to 50% of cardiac output is due to shunts in ARDS (high V/Q areas). The amount of blood flow is directly related to the degree of hypoxemia in patients [[68], [69], [70]].

These V/Q regions can change suddenly from high to low, and vice versa, if increased cardiac output is not followed by proportional alveolar ventilation [70]. Increased cardiac output has been observed in patients admitted to intensive care units with impaired alveolar ventilation due to protective pulmonary ventilation strategies and an inflammatory state [70,71]. For this reason, the root knowledge of gas exchange disorder in ARDS induced by COVID-19 becomes relevant when employing a therapeutic strategy for reasons that will be addressed later [72].

It is relevant to note that there are significant differences in perfusion, shunt, and ventilation-perfusion in the models studied, secondary to the heterogeneity of alveolar units. The severity of hypoxemia in infected patients cannot be explained without extensive perfusion defects, mismatch V/Q, or overperfusion of non-oxygenated regions [73]. A small cohort study reported an average shunt fraction of 50% versus 17% in non-ventilated lung units [73,74]. The ratio of shunts to non-ventilated pulmonary units was three; the fraction of shunts reported in other forms of ARDS was estimated at 1.25 ± 0.8 [62,[71], [72], [73], [74]]. These differences in poorly ventilated areas suggest deterioration in the hypoxic pulmonary vasoconstriction (HPV) mechanism. In other words, this mechanism induces pulmonary arteriolar constriction in response to impaired regional oxygenation, and subsequent failure leads to a significant mismatch in local ventilation and perfusion. The associated coagulopathy and secondary microthrombosis, which redirect perfusion to pulmonary regions with a low or no V/Q ratio, should not be forgotten [62].

**Fig. 5:**
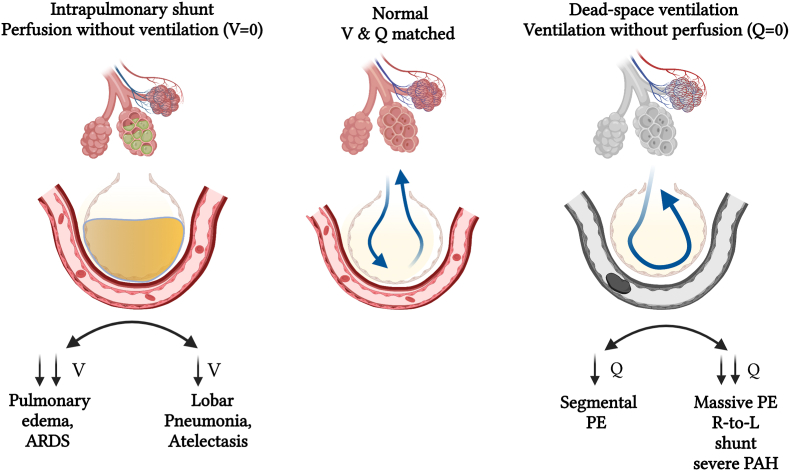
Spectrum of ventilation/perfusion (V/Q) mismatch in COVID-19. The coexistence of One or more abnormalities in injured alveoli may be involved in patients with COVID-19. As previously stated, direct or indirect damage leads to the release of inflammatory cytokines and reactive oxygen species (ROS) release, leading to impaired paracellular permeability and alveolar edema that disrupt ventilation. The prothrombotic effects of viral infections cause impaired perfusion, which leads to severe hypoxia. Thus, both events can coexist. The impact of mechanical ventilation associated with PEEP use, especially in patients with poor lung elasticity leading to lung overdistention and collapse of relatively preserved alveolar units, should not be overlooked and should further contribute to the ventilation-perfusion impairment seen in this patient population.

Diffusion is also limited when inflammation and edema impair the exchange barrier (blood gas), worsening hypoxemia [62] (Fig. 5).

The normofunctional HPV mechanism generates lower fractions of shunts and limited hypoxemia, which prevents a partial pressure of oxygen (PaO_2_)/FiO_2_ <300 mmHg (62). The models analyzed have estimated that a limited fraction of the injured lung between 0 and 30% with an absent exchange mechanism and vasodilation in the injured regions are determining factors for PaO_2_/FiO_2_ <300 mmHg, and have demonstrated that the HPV mechanism by itself does not sufficiently explain the observed levels of hypoxemia [62,74]. A reduction of up to 70% in pulmonary vascular resistance is required to explain the large shunts without a V/Q mismatch in a perfused-injured lung. This level decreases as the perfusion defect increases [74].

Concentrating the lesion in dependent lung areas generates higher shunt fractions and even more marked hypoxemia. A lower PaO_2_/FiO^2^ ratio is expected in patients with short circuits at high oxygen administration; however, the shunts are insensitive to an increase in FiO_2_ [74].

Interesting are the findings published by Herrmann et al. [62] that demonstrated the calculated fraction of shunts was three times higher for a relatively small fraction of injured, poorly ventilated lungs. The authors suggested that this disproportion involves extensive perfusion defects, perfusion defects combined with V/Q mismatch in the uninjured lung, and overflow of the small injured fraction [62]. Swenson et al. [75] have shown that variations in the mechanism of hypoxic pulmonary vasoconstriction vary up to five-fold between individuals and pulmonary regions, and are noted with decreases in FiO2 as small as 0.15 to 0.18. These variations can give rise to persistent pulmonary vascular resistance under inflammatory conditions and activation of proliferative pathways. It was established days to weeks after the initiation of these processes. The increase in pulmonary arterial pressure secondary to the HPV mechanism was not reversed by reversing hypoxia. A moderate increase in pulmonary artery pressure could increase blood flow in pulmonary hypoperfusion areas and, consecutively, with an increase in the recruitment of the total alveolar-capillary surface area that improves gas exchange [75].

## 6 Conclusion

It is evident that none of the previously revealed conditioning factors individually explain the effects on the silent hypoxia characteristic of COVID-19, which is reasonably better explained by the combined impact of all these factors. The importance of understanding the pathophysiological aspects previously exposed better explains the constellation of signs and symptoms that characterize COVID-19 infection and that differ significantly from other viral diseases that cause acute respiratory distress syndrome. Directing and focusing on therapeutic strategies can undoubtedly lead to therapeutic success.

## Future Perspectives

The pandemic secondary to the SARS-CoV2 virus has highlighted the weaknesses of health and epidemiological systems worldwide. The outbreaks of the infection will continue, and the long-term effects, mainly focused on the most critical organ systems, are still being fully understood. The constant increase in the subvariants continues, although with less intensity and lower mortality, especially in sectors that already have access to vaccination; however, millions of people worldwide still live in uncertainty. The knowledge based on the pathophysiological aspects of the disease must continue as part of the strategy to develop effective medications that block the viral interaction with these routes of entry. Priority should be given to efforts to continue developing effective vaccines and vaccination schedule intervals for a disease with endemic characteristics. The study of new technologies focused on nanoparticles, and bioflavonoids, currently little studied and with antiviral properties, should be initiated to expand the therapeutic arsenal for this and other viral pandemics, which undoubtedly endanger humanity daily.

## Human subjects/informed consent statement

Not applicable.

## Sources of funding

No funding

## Data availability statement

Not applicable.

## Provenance and peer review

Not commissioned, externally peer reviewed.

## Please state any conflicts of interest

The author declares that there is no conflict of interest.

## Please state any sources of funding for your research

No funding

## Ethical approval

Not applicable.

## Consent

Not applicable.

## Author contributions

This manuscript was written entirely by this author.

## Registration of research studies

1. Name of the registry: Not applicable
2. Unique Identifying number or registration ID: Not applicable
3. Hyperlink to your specific registration (must be publicly accessible and will be checked): None

## Guarantor

Dr. Francisco Javier González Ruiz.

## Declaration of competing interest

The author declares that there is no conflict of interest.
